# Chronic co-implantation of ultraflexible neural electrodes and a cranial window

**DOI:** 10.1117/1.NPh.9.3.032204

**Published:** 2022-01-07

**Authors:** Rongkang Yin, Brian C. Noble, Fei He, Pavlo Zolotavin, Haad Rathore, Yifu Jin, Nicole Sevilla, Chong Xie, Lan Luan

**Affiliations:** a Rice University, Department of Electrical and Computer Engineering, Houston, Texas, United States; b Rice University, Rice Neuroengineering Initiative, Houston, Texas, United States; c Rice University, Applied Physics Graduate Program, Houston, Texas, United States; d Rice University, Department of Bioengineering, Houston, Texas, United States

**Keywords:** flexible electrodes, cranial window, electrophysiology, optical imaging

## Abstract

**Significance:**

Electrophysiological recording and optical imaging are two prevalent neurotechnologies with complementary strengths, the combined application of which can significantly improve our capacity in deciphering neural circuits. Flexible electrode arrays can support longitudinal optical imaging in the same brain region, but their mechanical flexibility makes surgical preparation challenging. Here, we provide a step-by-step protocol by which an ultraflexible nanoelectronic thread is co-implanted with a cranial window in a single surgery to enable chronic, dual-modal measurements.

**Aim:**

The method uses 1-μm-thick polymer neural electrodes which conform to the site of implantation. The mechanical flexibility of the probe allows bending without breaking and enables long-lasting electrophysiological recordings of single-unit activities and concurrent, high-resolution optical imaging through the cranial window.

**Approach:**

The protocol describes methods and procedures to co-implant an ultraflexible electrode array and a glass cranial window in the mouse neocortex. The implantation strategy includes temporary attachment of flexible electrodes to a retractable tungsten-microwire insertion shuttle, craniotomy, stereotaxic insertion of the electrode array, skull fixation of the cranial window and electrode, and installation of a head plate.

**Results:**

The resultant implant allows simultaneous interrogation of brain activity both electrophysiologically and optically for several months. Importantly, a variety of optical imaging modalities, including wide-field fluorescent imaging, two-photon microscopy, and functional optical imaging, can be readily applied to the specific brain region where ultraflexible electrodes record from.

**Conclusions:**

The protocol describes a method for co-implantation of ultraflexible neural electrodes and a cranial window for chronic, multimodal measurements of brain activity in mice. Device preparation and surgical implantation are described in detail to guide the adaptation of these methods for other flexible neural implants and cranial windows.

## 1 Introduction

The brain has an enormous dynamic range spatially and temporally. Temporally, moment-by-moment information is processed at milliseconds,^1^ but changes in activity patterns that underlie adaptation, learning, development, and degeneration occur on broader timescales ranging from seconds to years and even decades.^2^^–^^4^ Spatially, neural activity involves not only cellular and subcellular structural and functional changes, but also orchestrated activities distributed across multiple brain areas. In addition to neuronal activity, brain functions and dysfunctions also stem from the interaction between other cell types and surrounding vasculature.^5^^,^^6^ Resolving these multifaceted activities requires technologies with cross-brain coverage, long-lasting functioning periods, and high spatial and temporal resolutions that match the network in question.^7^ Electrophysiology, one of the “gold standard” tools in detecting neuronal dynamics, affords high temporal resolution detection and direct neural activation, but is limited by its low spatial resolution incapable of resolving fine cellular and subcellular structures. In contrast, optical imaging and modulation techniques offer high spatial resolution^8^^,^^9^ and cell type specificity,^10^ and can measure non-neuronal, non-electrical activities.^11^^,^^12^ Nevertheless, they are limited by their generally low temporal resolution and insufficient tissue penetration depth. Integrating the electrical and optical measurements in the same brain leverages their complementary strengths, which has become an emerging approach for synchronously observing and controlling brain activities of a specific region.^13^^–^^18^

**Fig. 1:**
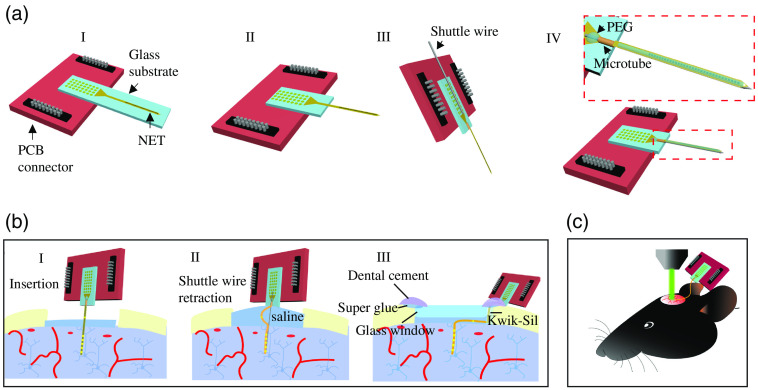
Schematics showing the overall workflow and outcome. (a) Key steps of NET preparation for implantation, including releasing of the flexible section (I, II) and temporary attachment of a shuttle wire (III, IV). (b) Key steps in cranial surgical procedures, including insertion of NET (I), retraction of the shuttle wire (II), and cranial window installation (III). (c) Post-surgery mouse supporting chronic, multimodal measurements that combine electrophysiology and optical methods.

Among various designs of neural electrodes, flexible electrodes^15^^,^^19^^,^^20^ are uniquely well-suited for concurrent, closely opposed optical and electrophysiological data collection. The mechanical flexibility allows these electrodes to be implanted beneath a cranial window and bent out of the way at a small radius without breaking or obstructing the optical view.^15^^,^^16^^,^^20^ Furthermore, and importantly, their mechanical flexibility alleviates mechanical micromotions^21^ and foreign-body responses^22^^–^^27^ at the tissue–electrode interface, which enables long-lasting recordings and stability to track individual neurons.^15^^,^^28^^–^^30^ However, the same mechanical flexibility makes surgical implantation a challenge. Here we use a 1-μm-thick, ultraflexible nanoelectronic thread (NET)^15^ to provide step-by-step guidance for surgical co-implantation of flexible electrodes and a cranial window in a single procedure. We include not only surgical procedures, but also preparation of ultraflexible electrodes for implantation and attachment of retractable shuttle devices (Fig. 1). To facilitate the adaptation of this method to other flexible electrodes, we use mostly off-the-shelf components, including cover glass as the cranial window, tungsten microwires as the shuttle devices, and polyetheretherketone (PEEK) microtubes as the guiding structures for shuttle devices. The same procedures can also be adapted to implanting other customized cranial windows of different sizes and materials.

## 2 Protocol

This protocol starts with pre-surgery device preparations for which a single-shank 32-channel NET electrode array is temporarily attached to an insertion shuttle made of a tungsten microwire. It proceeds with descriptions of surgical techniques and implantation steps required to successfully implant both the NET array and a cranial window in a mouse (Fig. 1). It ends with representative electrophysiological recordings and optical imaging from the co-implantation of NET and a cranial window.

**Fig. 2:**
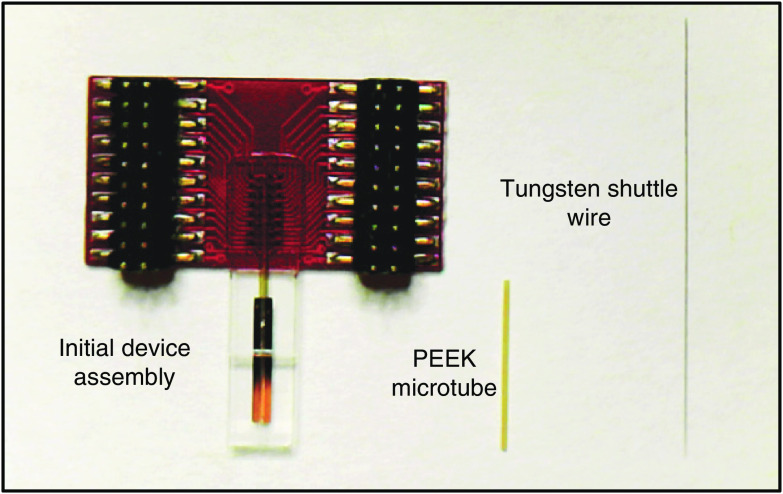
Insertion shuttle assembly components: an ultraflexible NET device before releasing from the fabricate substrate; a 10 to 15 mm long 50-μm diameter tungsten wire shuttle, and a PEEK capillary tube (O.D. 300  μm, I. D. 75  μm) for alignment and insertion.

This protocol assumes the starting materials of a NET array before releasing from the fabrication substrate of glass, a tungsten microwire (diameter: 50  μm, length: 10 to 15 mm) as the insertion shuttle, the bio-dissolvable adhesive polyethylene glycol (PEG) as the temporary adhesive,^31^ and a PEEK microtube as the guiding structures for the insertion shuttle (Fig. 2). The pre-released NET array is bonded with a customized printed circuit board (PCB) by a standard ball grid array packaging method, in which solder balls are manually placed on the bonding pad of NETs and brought into contact with the PCB that has matching copper pads. The assembly is then heated in a reflow oven to melt the solder balls and make a permanent bonding. After bonding, epoxy is applied at the edge of the NET device where it meets the PCB as mechanical reinforcement. The tip of the tungsten shuttle is pre-sharpened using KOH electrochemical etching as previously reported.^32^ This protocol also uses a #1 cover glass (diameter: 3 mm) and a 3D-printed piece to securely attach the NET device to a stereotaxic micromanipulator. All animal-involved protocols described in this protocol have been approved by the Institutional Animal Care and Use Committee at Rice University.

### 2.1 Preparation of Ultraflexible NET for Implantation

#### 2.1.1 Assembly of PEEK tube and tungsten shuttle

**Fig. 3:**
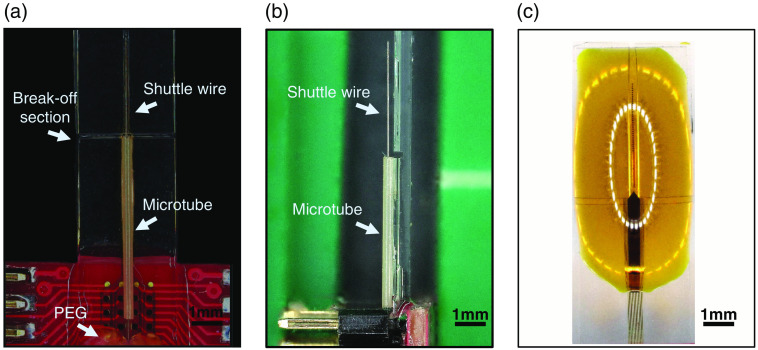
Procedures of mounting a PEEK tube and a tungsten shuttle, and releasing of NET from the substrate. (a) Front view and (b) side view of the tungsten shuttle feeding through a PEEK tubing. (c) The flexible section of NET immersed in Ni etchant to remove the Ni sacrificial layer.

1. Clean and rinse the NET device in isopropanol, and blow dry.
2. Use a pair of tweezers to attach the PEEK tube near the base of the NET bonding pad [Fig. 3(a)]. The PEEK tube is ∼6-mm long, extending from the bottom of the bonding pad. Add a small amount of adhesive epoxy on the outside of the tube to fully fix its position and secure it to the NET carrier chip.
3. Retrieve the sharpened tungsten-wire shuttle using a pair of forceps or micro-pincers. Grip and slowly guide the wire shuttle through the PEEK tube. Gently push the wire forward and toward the tip of the NET electrode. Keep driving the shuttle wire through the tube and across the glass surface until reaching the end of the device. The shuttle should stop ∼300 to 400  μm before reaching the tip of the unreleased NET [Figs. 3(a) and 3(b)].
4. Apply PEG (8 k molecular weight) as the dissolvable adhesive between the tungsten microwire and the PEEK tube. This step will fix the tungsten shuttle to its current position, preventing any unintended movements during future handling and insertion.
5. Remove the sacrificial layer Ni on the NET device to release the flexible section of NET by applying Ni etchant for ∼10 to 30 min [Fig. 3(c)]. Once all the Ni is etched away and the device is released, the device will float in the Ni etchant. Wash away any excess Ni etchant using deionized (DI) water. Keep the released NET section in DI water before attaching it to the shuttle wire.

#### 2.1.2 Alignment of NET thread to the insertion shuttle

**Fig. 4:**
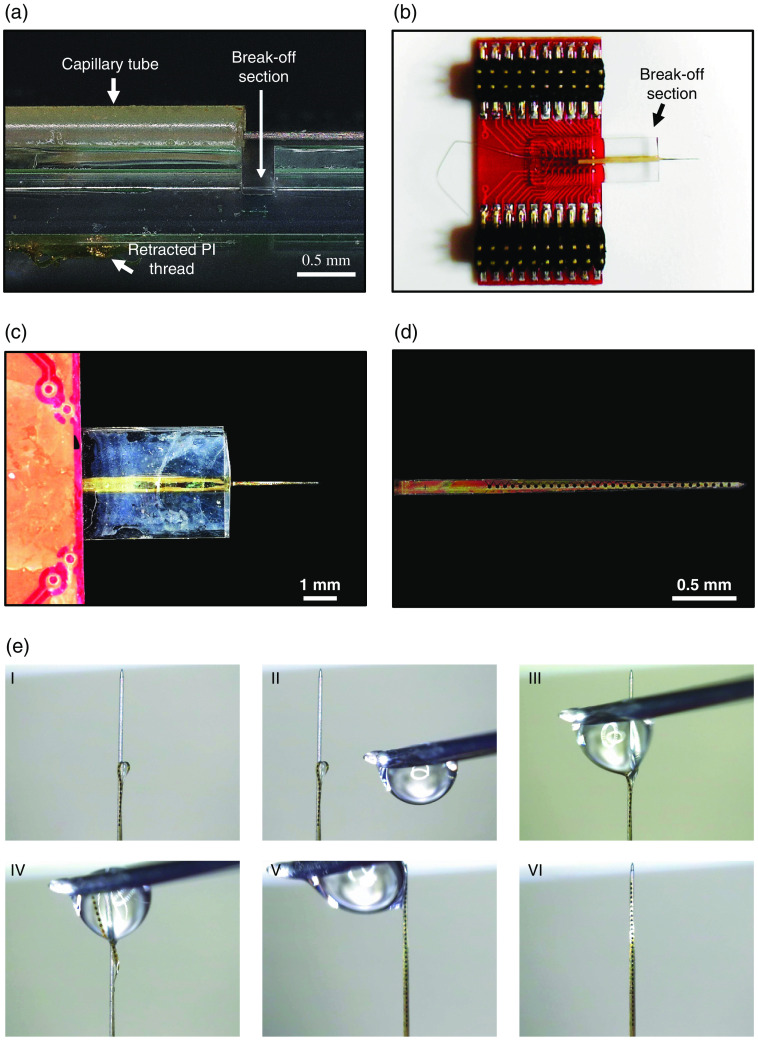
Procedures of aligning a NET to an insertion shuttle. (a) Zoom-in side view of a NET device showing the relative position of the PEEK tube, the tungsten shuttle, and the retracted flexible section of NET before cleaving off a section of the substrate. (b)–(d) NET-shuttle ensemble overview (b, c) and zoom-in view of NET aligned on the tungsten shuttle. (e) Snapshots showing the NET-shuttle alignment procedures that leveraged the surface tension of a water droplet.

1. Immerse the released device in DI water. Use a fine needle and carefully place the flexible NET section at least 0.5 mm away from the cleaving mark on the carrier chip [Fig. 4(a)].
2. Break off the bottom piece of glass by applying concentrated pressure on the cleaving mark using a pair of wafer cleaving/glass breaking pliers [Fig. 4(b)].
3. Manually align the ultraflexible section of NET onto the tungsten wire shuttle [Figs. 4(c) and 4(d)]. Keep the device fixed in place using adhesive tape. Leverage the surface tension of water to wrap the ultraflexible NET onto the tungsten wire. Use a 31-gauge needle to control a single drop of water and the associated water-air surface tension to align the electrode thread on the tungsten shuttle [Fig. 4(e) and supplementary video]. The alignment between the flexible section of NET and the shuttle wire needs to satisfy the following criteria: (a) The individual recording sites on NETs face away from the tungsten wire so they will be in intimate contact with the brain tissue during and after implantation. (b) The tip of the NET thread should not extend beyond the tip of the tungsten wire to decrease the chance of premature detachment during implantation.
4. Once the flexible section of NET and the shuttle wire is appropriately aligned, apply 10% PEG aqueous solution using a 31-gauge needle to attach NET with the wire. We choose PEG because PEG is biocompatible and biodegradable. The dissolution time by the cerebrospinal fluid is a few minutes, which is conveniently short for prompt implantations, and sufficiently long to support implantations into mouse cortex and hippocampus. Start at the tip of the shuttle device and work along the thread to the end of the flexible section. The thickness of the PEG adhesion layer should not exceed two microns^32^ and therefore not easily visible under the microscope [Fig. 4(d)].
  Note: If the flexible device is placed too short or too long from the sharpened end of the shuttle, adjust the wire’s position by first dissolving the PEG attaching the shuttle to the PEEK tube. Reposition the shuttle using a pair of tweezers to the proper position and reapply PEG to re-fix its location.
5. Sterilize the assembled device by ethylene oxide low-temperature sterilization. The total weight of the implant is <1  g.

### 2.2 Surgical Procedures for Co-Implantation of NET and a Cranial Window

#### 2.2.1 Craniotomy

**Fig. 5:**
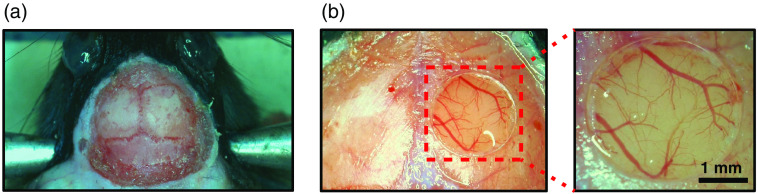
Preparation of the skull and craniotomy. (a) An animal with scalp removed and skull prepared for craniotomy. (b) A 3-mm diameter cranial window opened above the somatosensory cortex. A zoom-in image shows minimal vessel damage and a clear 3-mm glass window securely fitting within the craniotomy.

1. Anesthetize the animal using isoflurane (3% for induction and 1% to 2% for maintenance). Place the animal on a homeothermic blanket at 37°C to maintain body temperature stable. Monitor the depth of anesthesia by periodic toe-pinch tests (every 15 min) and the breath rate (55 to 65  breaths/min).
2. Administer Ethiqa-XR (3.25  mg/kg) for analgesia and dexamethasone (2  mg/kg, subcutaneous) for anti-inflammation after induction. Apply ophthalmic ointment on the eyes to prevent drying of the cornea. Administer warmed lactated ringers subcutaneously 1  ml/100  g body weight per anesthesia hour to minimize postoperative dehydration.
3. Shave fur on the surgical site with a clipper.
4. Fix the mouse on the stereotaxic apparatus. Position the intra aural positioning studs to symmetrically fix the position of the animal’s head in the apparatus. Use only blunted style ear studs for survival procedures. To insert the incisor adapter, use small forceps to pull down the animal’s lower jaw and slowly move the incisor adapter into the mouth until the incisors fit in the opening; gently pull back slightly and fix the adapter in place. Gently position the nose clamp by resting on top of the nose using very low pressure. Adjust the incisor screw to make the head level so that bregma and lambda are equal in height. Level the head horizontally in the caudal-to-rostral direction.
5. Sterilize the surgical site via alternating (3×) scrubbing of iodine (betadine) and 70% isopropyl alcohol. Administer 0.5% Lidocaine (7  mg/kg) underneath the scalp.
6. Incise into the skin of the scalp and resect to expose the skull between bregma and lambda skull sutures [Fig. 5(a)]. Remove the periosteum and score the skull with the tip of a scalpel blade to roughen the skull.
7. Measure the desired coordinates with a pair of calipers and use a scalpel blade to mark the position. Thin the bone along the marked craniotomy perimeter using a surgical drill. Drill a circular craniotomy with a diameter of 3 mm over the target brain areas [Fig. 5(b)].
  Note: Regularly rinse the skull with sterile saline during drilling to prevent heating. The thickness of the bone along the drilled perimeter can be checked by gently pressing on the bone. Drill until the central piece of the bone is detached from the rest of the skull.
8. Using a pair of forceps, carefully lift the central piece of the skull. If dura is attached to some part of the skull, apply sterile saline, slowly pry the edge, and follow around.
9. Immediately after removing the central piece of the skull, place a piece of saline-soaked gel foam in the craniotomy to keep the exposed brain moist and promote coagulation.
10. Carefully remove any remaining thin layer of bone on the edge of the craniotomy. This prevents rapid bone regrowth under the window that impedes optical clarity for chronic studies. Remove small residues of bone dust and blood clots.
11. Drill a burr hole at the contralateral hemisphere of the brain and insert a metal wire (made of platinum, silver, or medical-grade stainless steel) as the grounding reference.

#### 2.2.2 Implantation of NET

**Fig. 6:**
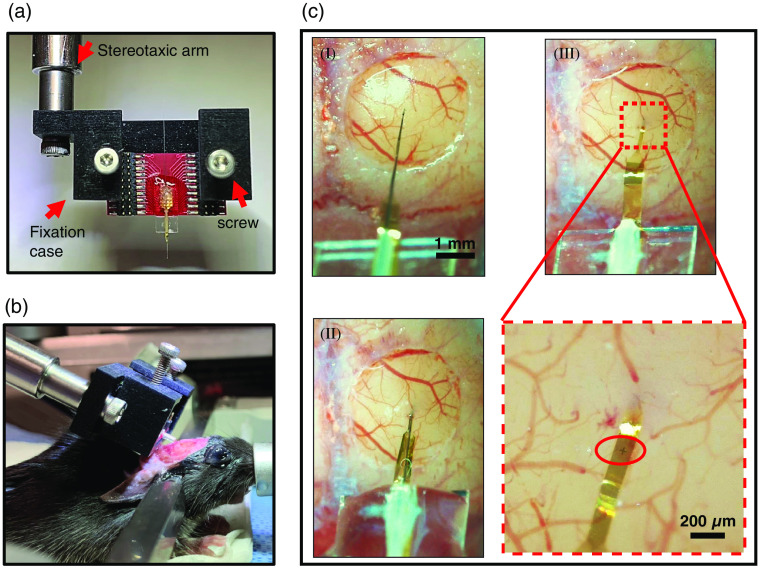
Intracortical implantation of NETs. (a) A NET device mounted on a stereotaxic arm with a 3D-printed fixation case. (b) Side view showing the mounting angle of the carrier chip to minimize interference with imaging objectives. (c) Implantation of the NET device. (I) Target the brain region of interest and avoid blood vessels. (II) Fast insertion of the NET with the tungsten shuttle. Dissolve PEG with saline to release NET from the shuttle. (III) Retraction of the tungsten shuttle and fine adjustment of implantation depth. Zoom-in image shows the pre-designed “+” marker (red oval) on NET for implantation depth control.

1. Fix the assembled NET device onto a stereotaxic arm using a 3D-printed case and a pair of screws [Fig. 6(a)].
2. Remove any gel foam from the craniotomy. Aim the device to the insertion site. To avoid surface vasculature, move in z-direction toward the brain and fine adjust the implantation location [Fig. 6(b-I)].
  Note: Avoid prolonged periods of the device in close proximity to the brain before insertion. Water vapor and condensation from the brain surface may weaken the adhesion between the NET and the insertion shuttle, leading to premature detachment prior to or during insertion.
3. Insert the device quickly. For a thin layer (1 to 2  μm) of PEG as we demonstrate here, the insertion time should be within 5 s to avoid pre-mature separation between the shuttle wire and PEG. To achieve slower insertion speed, thicker layer of PEG can be used at the expense of larger implantation footprint. After insertion, dissolve PEG outside the brain by gently dripping body-temperature saline on the device where PEG is adhered to the shuttle needle. Wait until PEG outside the brain is completely dissolved (<10  min). The device will float in saline and separate from the shuttle needle, indicating it is ready for retraction [Fig. 6(b-II)]. By this time, PEG inside the brain should have also been dissolved by cerebrospinal fluid.
4. Grip the PCB end of the shuttle wire using a pair of tweezers and retract the shuttle wire manually. During retraction, carefully inspect the interface between the implanted NET and the shuttle needle through the surgical microscope. The implanted section of NET should not move with the shuttle wire.
5. To achieve precise implantation depth after shuttle needle is retracted, slowly retract the stereotaxic arm, straighten the flexible section outside the brain, and pull out the implanted section to reach the designated depth. Depth markers are fabricated on NET as guidance [Fig. 6(b-III)].
6. Rotate the stereotaxic arm to reduce the mounting angle of the carrier chip on the skull. Depending on the rotation axis, this may involve iterative steps of rotation and moving the micromanipulator on the stereotaxic arm. Avoid straightening the flexible section of NET outside the brain to minimize uncontrolled stress on it [Fig. 6(b-III)]. The carrier chip should be more than 2 mm away from the edge of the craniotomy and at an angle of 45 deg or shallower to accommodate the optical objectives for two-photon imaging [Fig. 6(c)]. The flexible section of NET outside the brain should have some slack (at least 500  μm) to accommodate possible tissue motion as initial implantation damage subsides with time. Fix the stereotaxic arm when the carrier chip reaches the final location and angle.

#### 2.2.3 Implantation of chronic cranial window

**Fig. 7:**
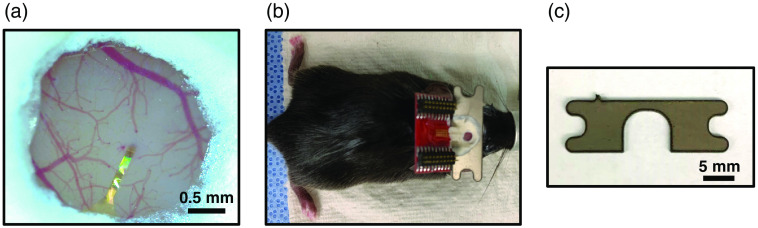
Co-implantation of the cranial window and a head-plate. (a) Installed cranial window on top of the NET. white shows the Metabond cement. (b) Overview of the headstage after implantation. NET chip carrier is mounted at an oblique angle. (c) The design of the titanium headbar for head-fixed, awake experiments.

1. After implantation of NET, add sterile saline to the brain. Place a glass window on the exposed area. If there is space between the window and the remaining skull, fill it with Kwik-sil.
2. Dry the skull. Apply a layer of low viscosity super glue on the remaining skull. Apply a layer of high viscosity super glue around the edge of the window, ensuring contact with the skull.
3. Apply a layer of Metabond dental cement over the high-viscosity super glue and the Kwik-sil, and apply additional cement to fix the NET carrier chip to the skull [Fig. 7(a)].
4. Mount a lightweight titanium headbar on the skull for head-restrained experiment by applying another layer of Metabond cement [Fig. 7(b)].
5. Cap the glass window using a piece of silicone to protect the window from mechanical scratches and tissue from photobleaching by ambient light.

#### 2.2.4 Postoperative care

Animals will be monitored and documented twice in the first 24 h post-surgery, then daily for four days on body weight, incisional appearance, posture and attitude (grimacing, squinting, walking on toes or hunched. Afterward, the mouse status (body weight; body condition, score as defined by Rice University’s “Monitoring Metric Scoring for Humane Endpoints to Score Animal Discomfort After Surgery” form; head cap condition) is documented weekly post-surgery.

### 2.3 Representative Results

**Fig. 8:**
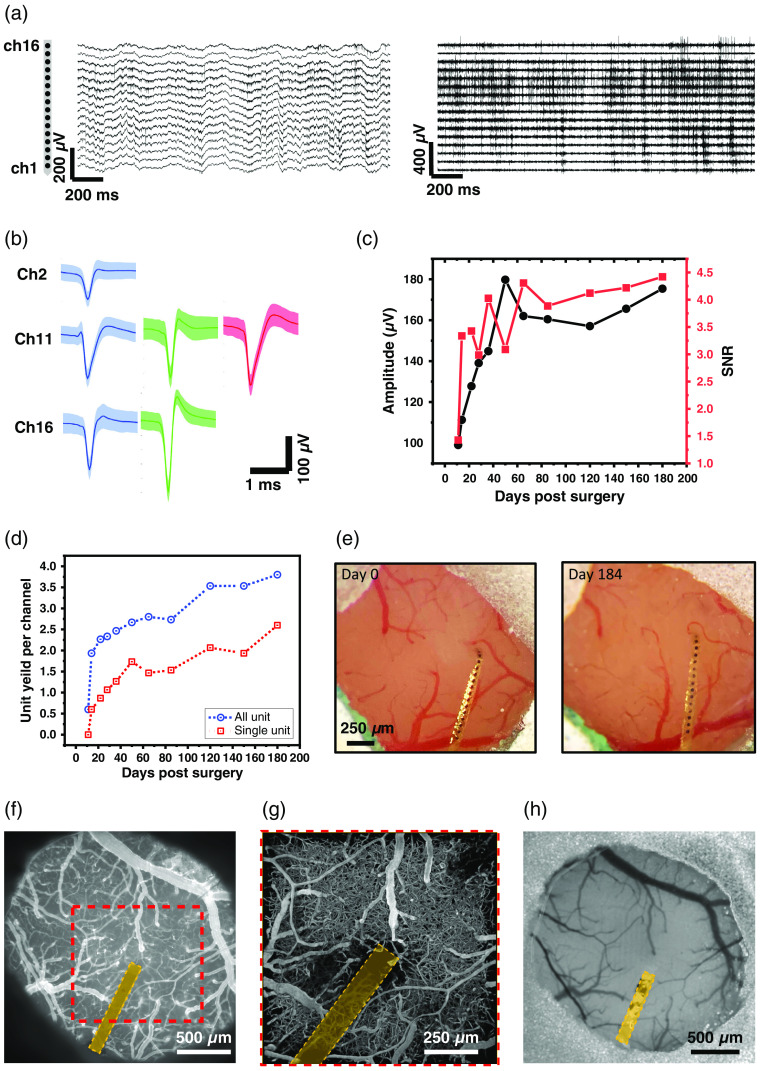
Representative results of closely opposed electrophysiological recordings and optical imaging of multiple modalities. (a) Unfiltered voltage traces (left) and band-pass (300–6000 Hz) filtered voltage traces (right) from 16 channels spanning the depth of the mouse cortex. (b) Single units isolated from three representative channels in (a). Solid line: average waveform; shade: standard deviation. (c) The averaged amplitude of all units and the averaged SNR of the longitudinal recordings in (a). (d) The all unit yield and single unit yield for all the channels of the longitudinal recordings. (e) Co-implanted cranial window at the day of surgery (day 0) and 184 days after surgery showing that the method affords long-lasting optical access. (f) Wide field fluorescence imaging of pial vessels. (g) Two photon imaging of vasculature from the dashed square in (f). Blood plasma was labeled by fluorescein isothiocyanate–dextran (FITC-dextran). Maximum intensity projection of a 600-μm-thick cortical microvascular stack is shown. (h) Laser speckle contrast imaging of cerebral blood flow surrounding the implanted NET. Orange band denotes NET in (f)–(h).

After this protocol, a 32-channel NET was co-implanted with a glass cranial window over the mouse motor cortex, resulting in closed opposed electrophysiological and optical data collection that lasted over 180 days. Figures 8(a) and 8(b) shows the representative recordings from 16 channels and representative units detected from the recordings at Day 180 after implantation. The unit yield, averaged amplitude of all units, and the signal-to-noise ratio (SNR) increased and fluctuated in the first 60 days, and remained stable for the rest period without any decay over the time course of 184 days [Figs. 8(c) and 8(d)]. Here “all unit” represents all sorted units from Mountain Sort after rejecting noise and artifact clusters.^33^ “single unit” represents the units that meet the single unit criteria: the number of spikes with an inter-spike interval smaller than 2 ms is <2% of the total events. The optical window remained clear without significant dura or bone regrowth for the same 6 month period [Fig. 8(e)]. In another animal with a co-implanted 32-channel NET and a glass cranial window over the somatosensory cortex, we showcased a variety of imaging modalities, including wide-field fluorescence imaging^34^ [Fig. 8(f)] and two-photon imaging of vasculature^9^ [Fig. 8(g)], and laser speckle contrast imaging of cerebral blood flow^35^ [Fig. 8(h)]. Mice were awake, head-fixed on a custom-made free-moving treadmill for these measurements. These results show the unique capabilities of the method described in this protocol in enabling longitudinal measurements of the multifaceted brain activity.

## 3 Discussion

This protocol provides step-by-step instruction on the implantation preparation of ultraflexible NET and its co-implantation with a cranial window, with the goal of disseminating these reproducible methods to the neuroscience community. While the protocol described here uses a single-shank NET as an example, the same procedures apply to multi-shank flexible electrodes as previously discussed.^32^

The methods use off-the-shelf, cost-effective components such as tungsten microwires and PEG, which is beneficial for their facile adaptation to other designs of flexible electrodes. One caveat associated with this method is the relatively low level of control in the dissolution time of PEG, resulting from the simple design of the shuttle device and adhesion mechanism. A more sophisticated design of the shuttle device, e.g., containing microfabricated reservoirs of PEG,^36^ can alleviate this problem. An alternative shuttle-device attachment mechanism, such as the needle and thread sewing mechanism,^15^ provides a promising approach to precisely control when and where the insertion shuttle detaches from the flexible electrodes.

## 4 Materials

Table 1 lists materials and relevant information.

**Table 1:** Materials list.

Name	Company	Catalog number	Comments
PEEK thin-wall tubing	Zeus	B00169K9NW	Guiding microtubes
Tungsten wire	Advent Research Materials	W560604	Diameter of 75 μm
Polyethylene glycol	Millipore Sigma	8188921000	Bioadhesive
			Molecular weight: 35000
Nickel Etchant Type I	Transene Company	012027	Nickel Etchant Type I contains metal chelating ingredients for optimal performance. Nickel Etchant Type I will not attack gold films.
Glass window	Warner Instruments	64-0720	No. 1 cover glass, diameter: 3 mm
Kwik-sil	World Precision Instruments	NA	Fill the gap between the skull and the cranial window
Super glue	Loctite	43903	Applied on Kwil-sil to promote the adhesion of Metabond cement to the glass window
Metabond cement	Parkell	s399	For sealing the cranial window and mounting the carrier chip to the skull
Kopf stereotaxic instrument	Kopf Instruments	Model 940	For mice
3D-printed pieces	NA	NA	Files available at: https://github.com/yinrocky/LuanLab3Ddesigns

## Code, Data, and Materials Availability

All data needed to evaluate the conclusions in the paper are present in the paper. Additional data related to this paper may be requested from the authors.
